# Supplementary figures and images for: Transmembrane protein western blotting: Impact of sample preparation on detection of SLC11A2 (DMT1) and SLC40A1 (ferroportin)
Source: PLoS One. 2020 Jul 9;15(7):e0235563. doi: 10.1371/journal.pone.0235563 (PMC7347119; doi:10.1371/journal.pone.0235563)

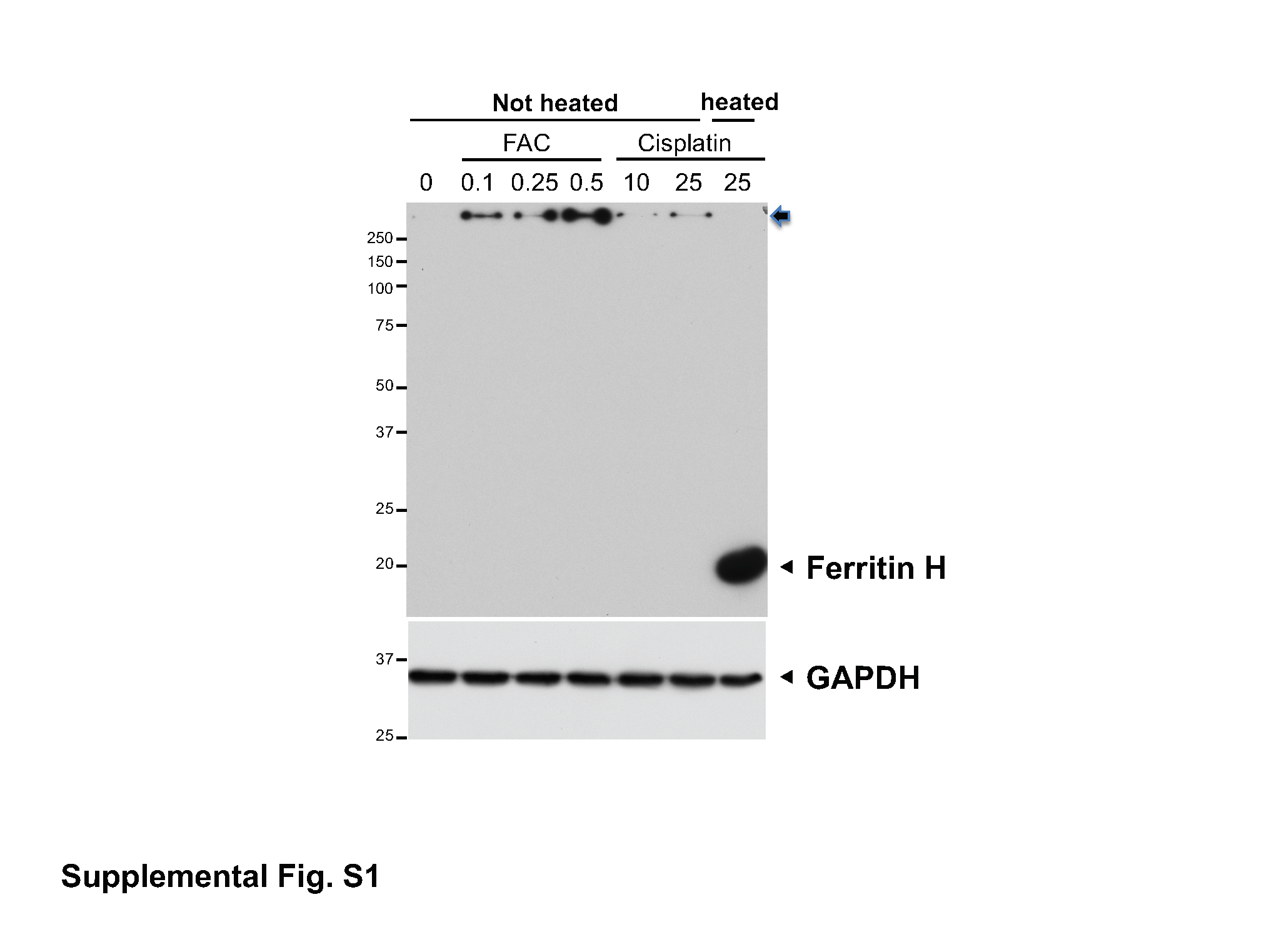

Supplement: S1 Fig — WCLs in RIPA buffer were prepared from SW480 cells treated with 0.1–0.5mM FAC (ferric ammonium citrate), 10 or 25ug/ml cisplatin for 18hr. 20ug of WCLs mixed with 2X SDSPAGE sample loading buffer without heating and 25uM cisplatin WCL without or with heating at 95°C, 5min were subjected to Western blotting with anti-ferritin H antibody, followed by incubation with anti-GAPDH antibody. The arrow indicates the ferritin protein stuck on the top of the separation gel. (TIFF) [file pone.0235563.s001.tiff]

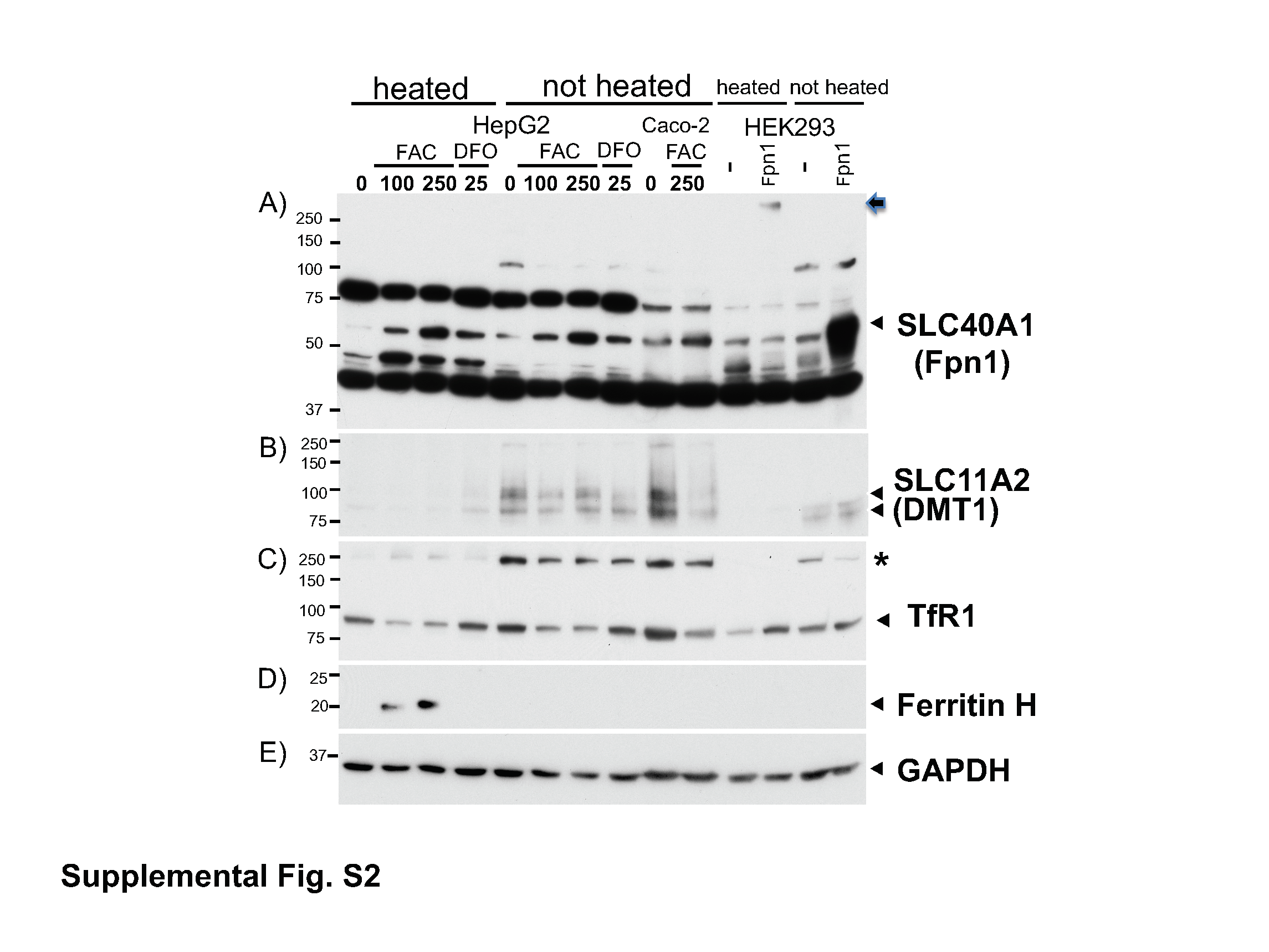

Supplement: S2 Fig — HepG2 cells were untreated or treated with 100, 250uM FAC, or 25uM DFO for 24hr. Caco-2 cells were untreated or treated with 250uM FAC for 24hr. 30ug of HepG2, Caco-2 WCLs along with 10ug of WCL from HEK293 cells transfected with pCMV or pCVM Fpn1 were heated at 95°C for 5min or not heated (at room temperature). They were subjected to successive western blotting with A) anti-Fpn1, B) anti-DMT1, C) anti-TfR1, D) anti-ferritin H, and E) anti-GAPDH antibodies. The arrow in A) indicates the transfected Fpn1 protein stuck on the top of the separation gel. The asterisk in C) may represent a TfR1 dimer. (TIFF) [file pone.0235563.s002.tiff]
