# Supplementary material for: Transmembrane protein western blotting: Impact of sample preparation on detection of SLC11A2 (DMT1) and SLC40A1 (ferroportin)
Source: PLoS One. 2020 Jul 9;15(7):e0235563. doi: 10.1371/journal.pone.0235563 (PMC7347119; doi:10.1371/journal.pone.0235563)

Fig. 1A

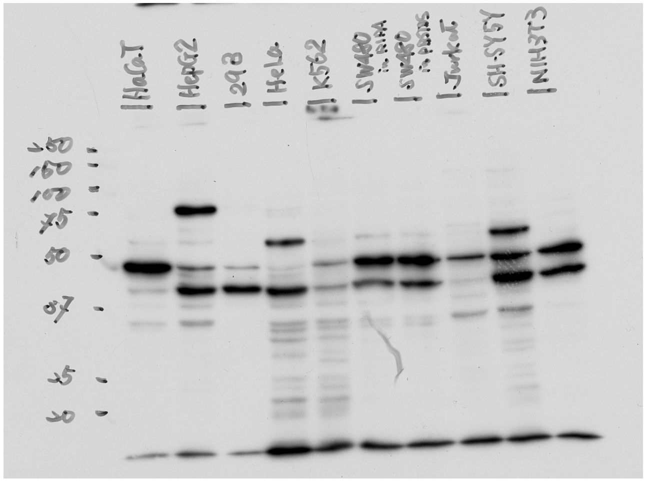

Fig. 1B top

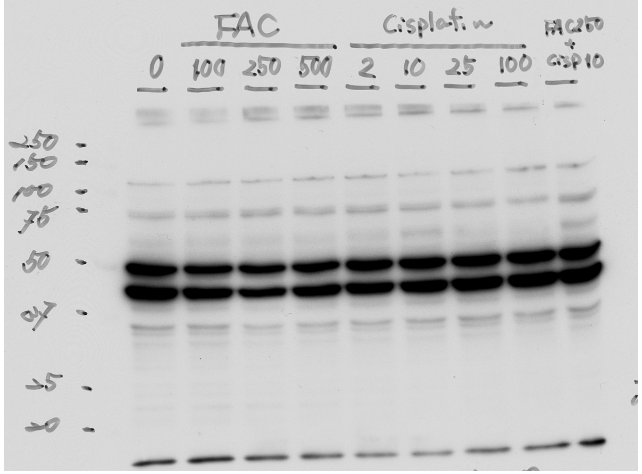

Fig. 1B bottom

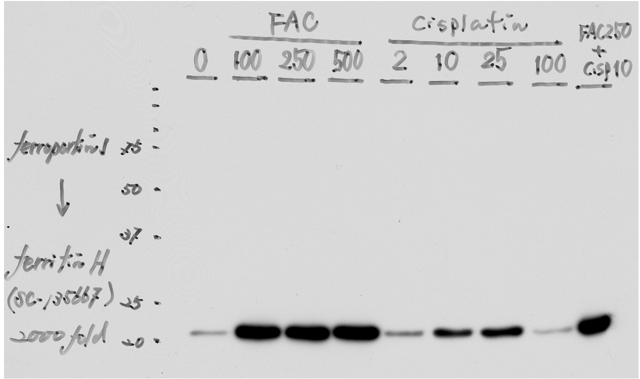

Fig. 2A

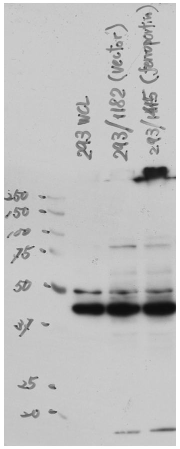

Fig. 2B

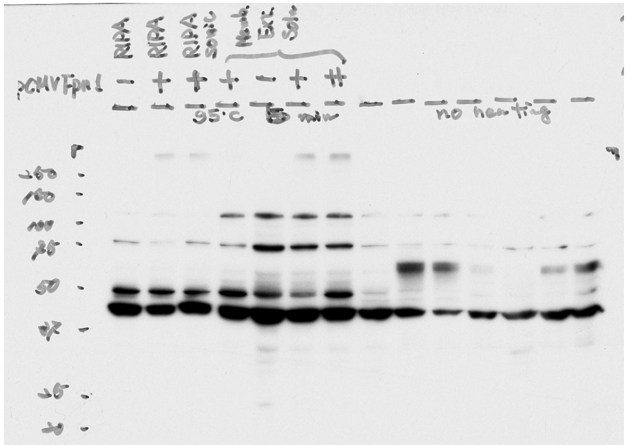

Fig. 2C

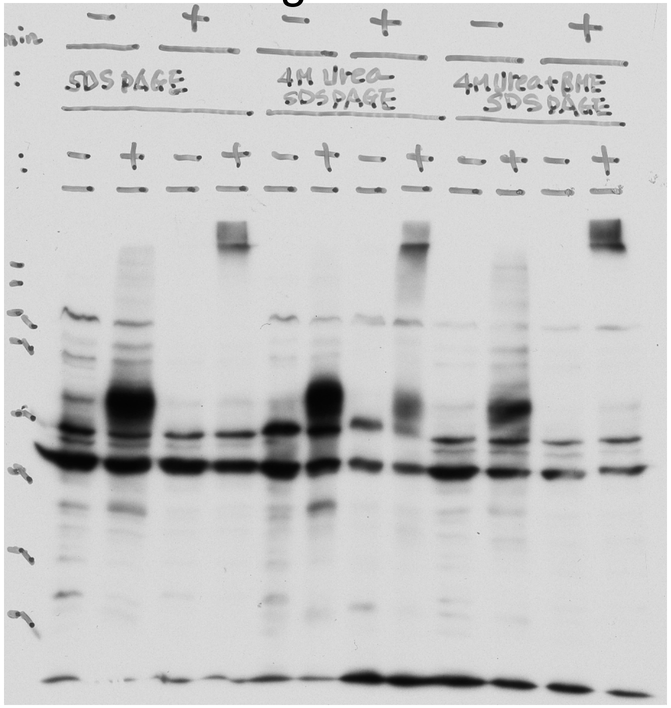

Fig. 3A

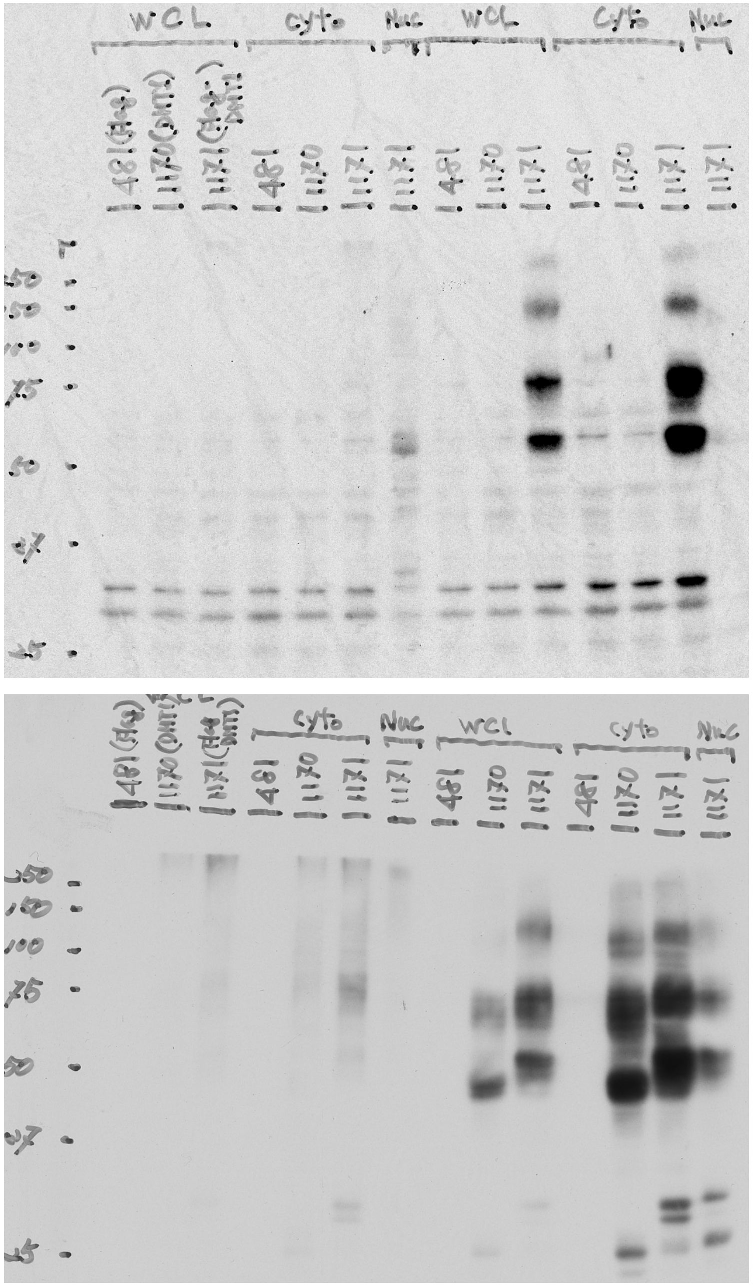
WCL
cyto
Nuc
WCL
cyto
Nuc
481
1170
1171
250
150
100
75
50
37
25

Fig. 3B

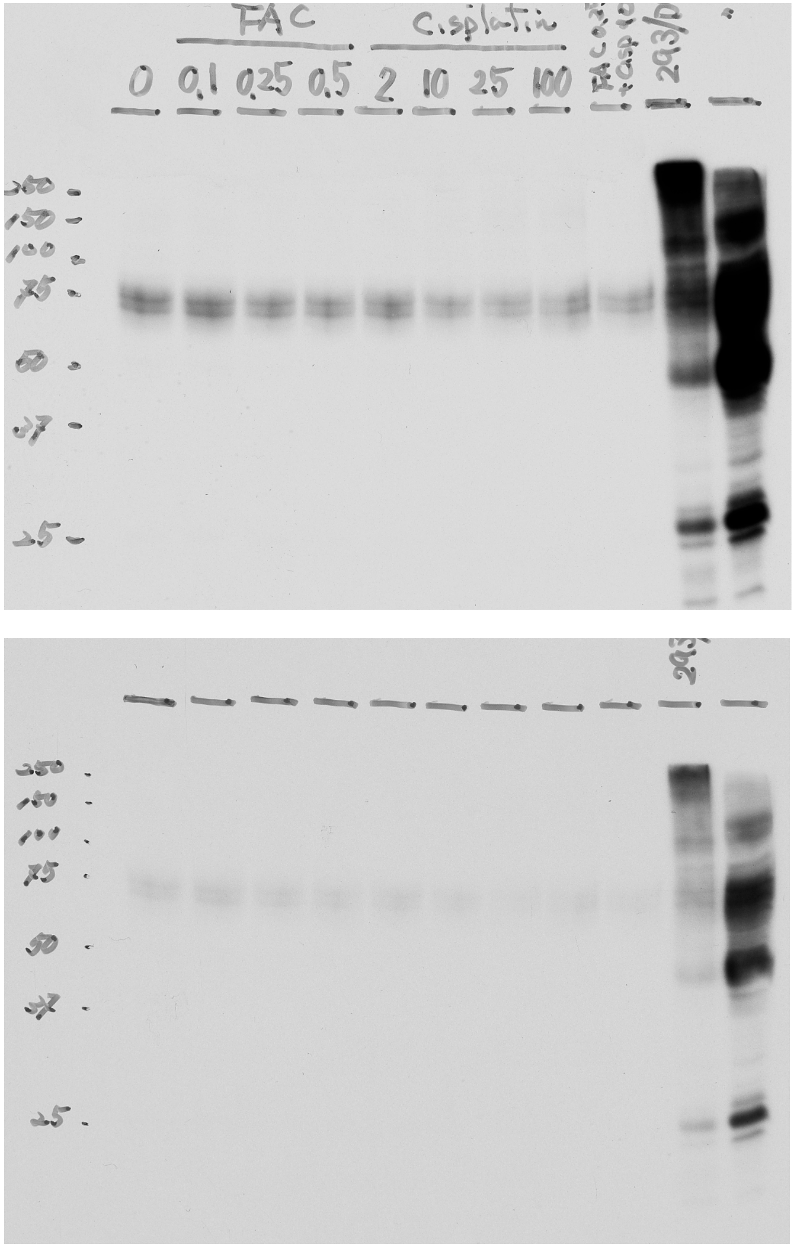
FAC
Cisplatin
0
0.1
0.25
0.5
2
10
25
100
293/D
250
150
100
75
50
37
25

Fig. 4A

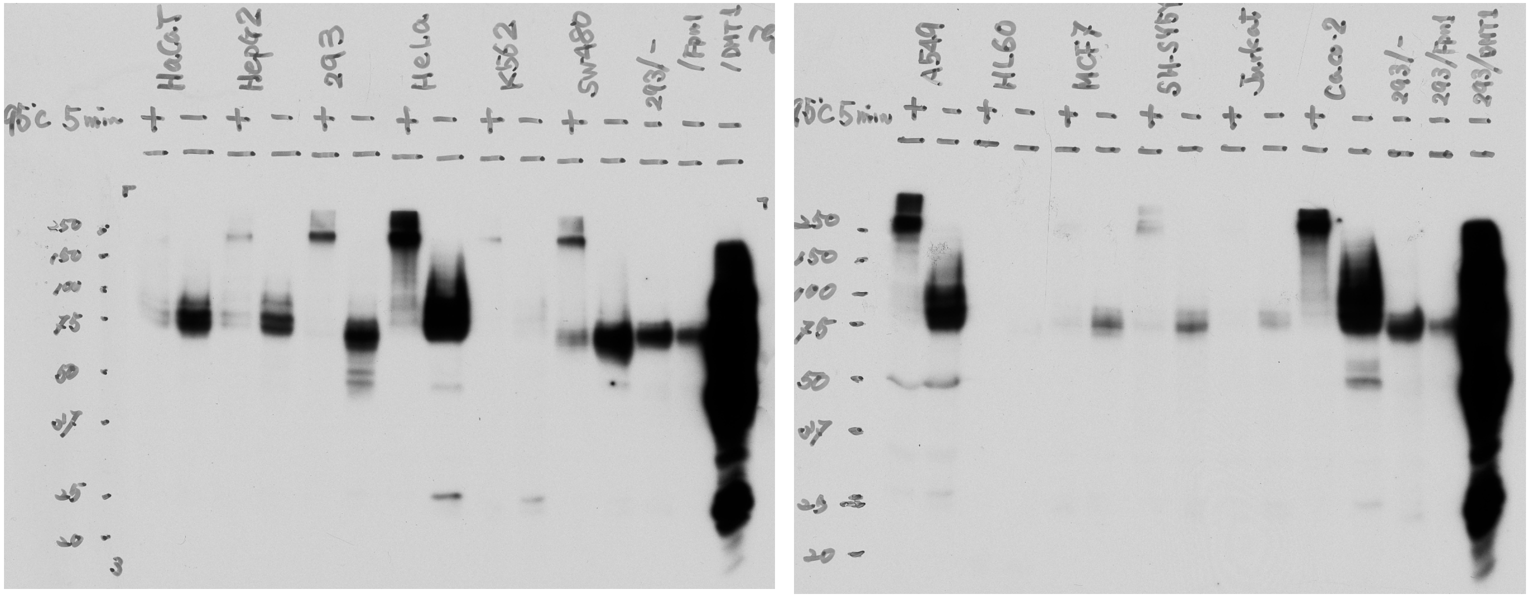
HaCaT
HepG2
293
HeLa
K562
SW480
293/-
/Fpn1
/DMT1
95°C 5min
A549
HL60
MCF7
SH-SY5Y
Jurkat
Caco-2
293/Fpn1
293/DMT1
250
150
100
75
50
37
25
20

Fig. 4B

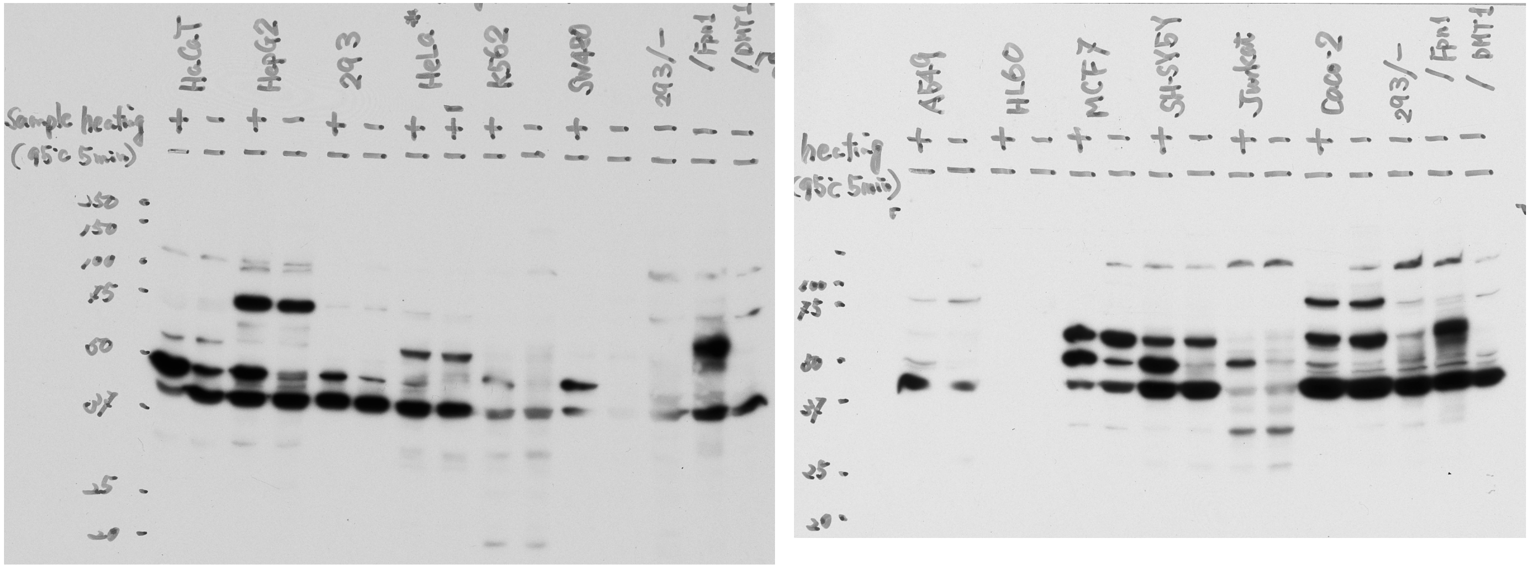
Sample heating
(95°C 5min)
HaCaT
HepG2
293
HeLa
K562
SW480
293/-
/Fpn1
/DMT1
heating
A549
HL60
MCF7
SH-SY5Y
Jurkat
Caco-2
250
150
100
75
50
37
25
20

Fig. 5A

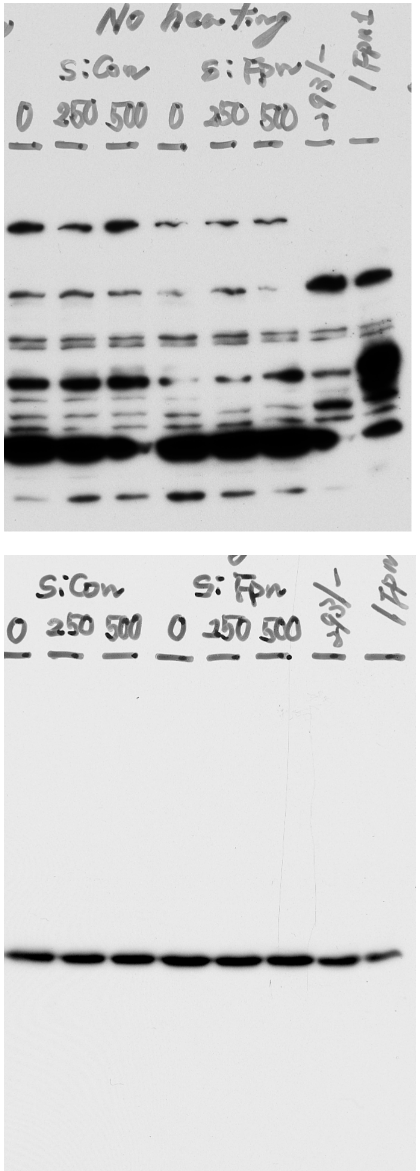

Fig. 5B

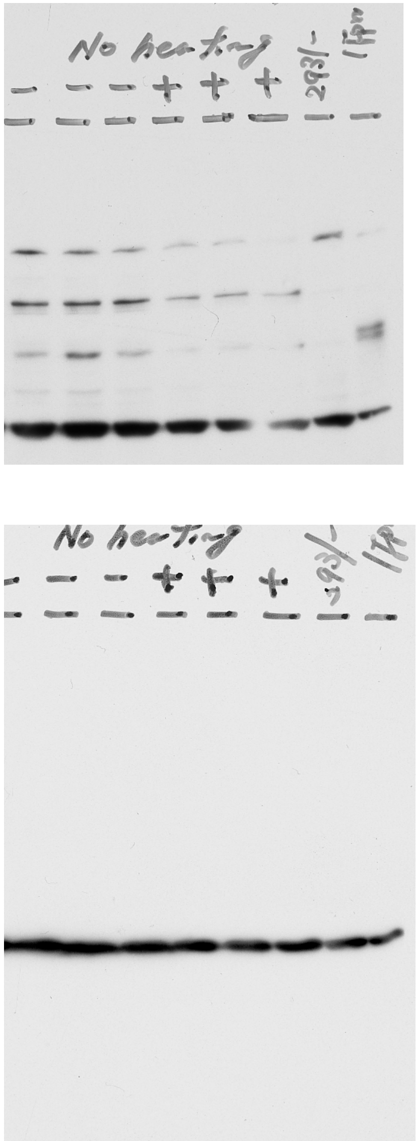

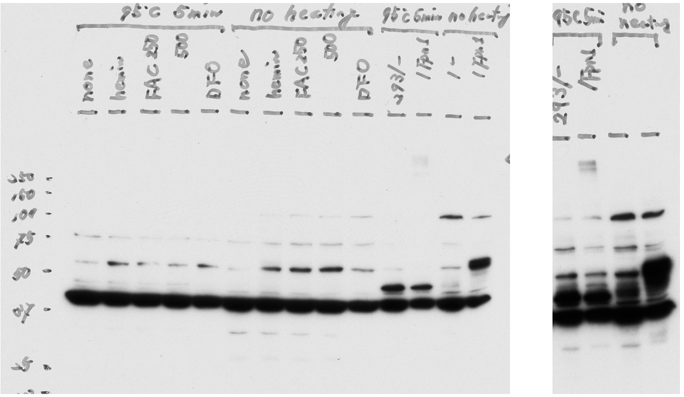

Fig. 6A

Fig. 6B

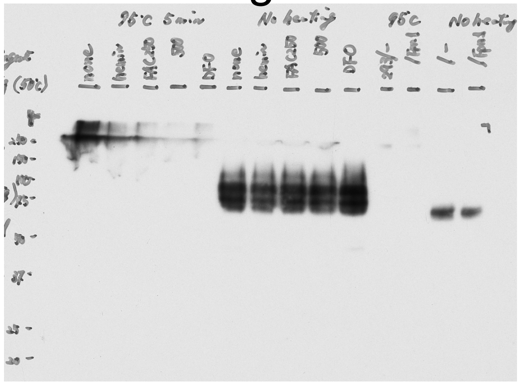

Fig. 6D

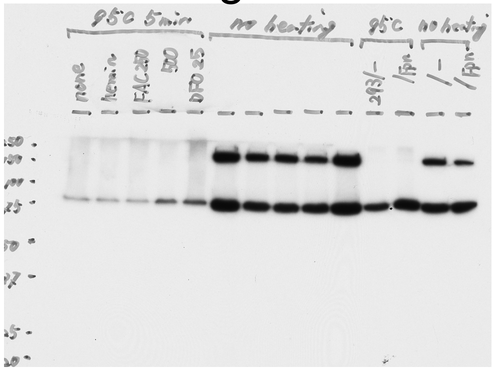

Fig. 6C

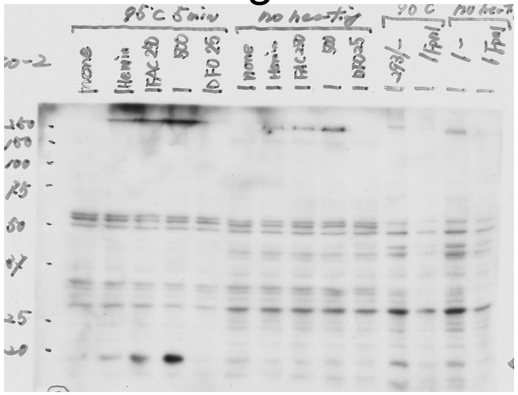

Fig. 6E

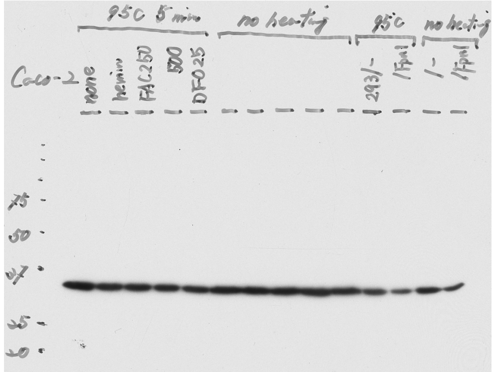

Fig. S1

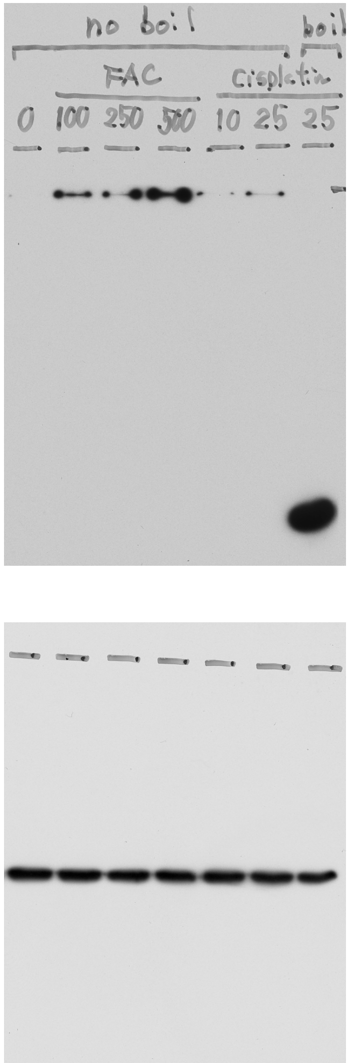

Fig. S2A

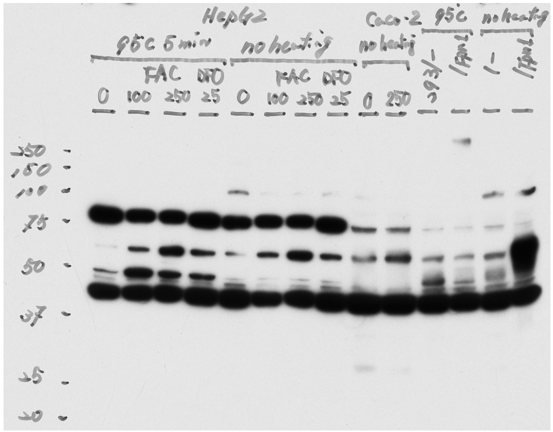

Fig. S2B

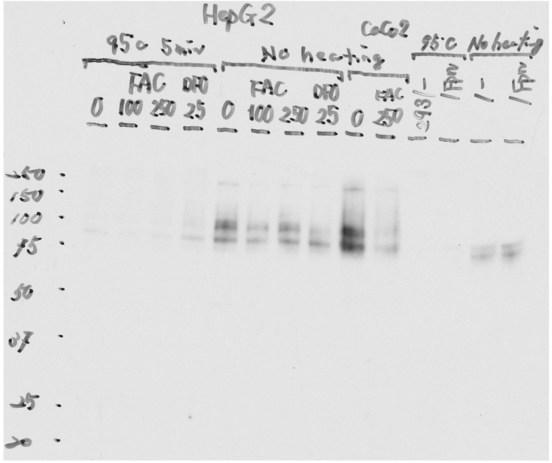

Fig. S2C

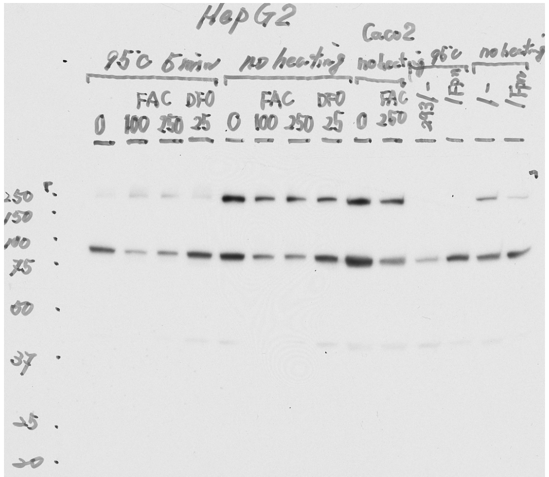

Fig. S2D

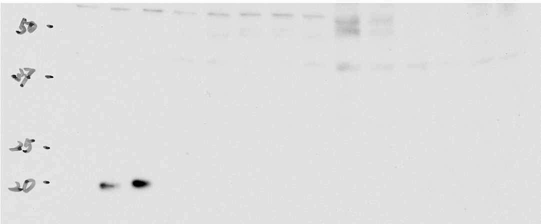

Fig. S2E

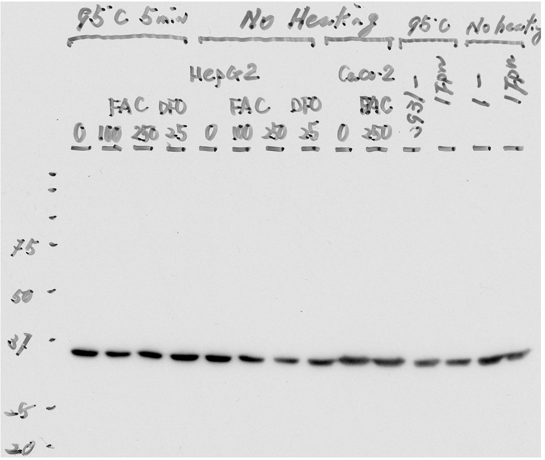

Supplement: S1 File — (PDF) [file pone.0235563.s003.pdf]
